# Consumption of Ultraprocessed Food and Risk of Depression

**DOI:** 10.1001/jamanetworkopen.2023.34770

**Published:** 2023-09-20

**Authors:** Chatpol Samuthpongtorn, Long H. Nguyen, Olivia I. Okereke, Dong D. Wang, Mingyang Song, Andrew T. Chan, Raaj S. Mehta

**Affiliations:** 1 Clinical and Translational Epidemiology Unit, Massachusetts General Hospital and Harvard Medical School, Boston; 2 Division of Gastroenterology, Massachusetts General Hospital and Harvard Medical School, Boston; 3 Department of Psychiatry, Massachusetts General Hospital and Harvard Medical School, Boston; 4 Department of Epidemiology, Harvard T.H. Chan School of Public Health, Boston, Massachusetts; 5 Channing Division of Network Medicine, Department of Medicine, Brigham and Women’s Hospital and Harvard Medical School, Boston, Massachusetts; 6 Department of Nutrition, Harvard T.H. Chan School of Public Health, Boston, Massachusetts; 7 Broad Institute of MIT and Harvard, Cambridge, Massachusetts; 8 Department of Immunology and Infectious Diseases, Harvard T.H. Chan School of Public Health, Boston, Massachusetts

## Abstract

This cohort study examines the consumption of ultraprocessed food and risk of depression among 31 172 US females aged 42 to 62 years.

## Introduction

Increasing evidence suggests that diet may influence risk of depression.^1,2,3^ Despite extensive data linking ultraprocessed foods (UPF; ie, energy-dense, palatable, and ready-to-eat items) with human disease,^4^ evidence examining the association between UPF consumption and depression is scant. Prior studies have been hampered by short-term dietary data^1,2^ and a limited ability to account for potential confounders.^1,3^ Additionally, no study has identified which UPF foods and/or ingredients that may be associated with risk of depression or how the timing of UPF consumption may be associated. Therefore, we investigated the prospective association between UPF and its components with incident depression.

## Methods

This cohort study was approved by the institutional review board (IRB) at the Brigham and Women’s Hospital and the Harvard T.H. Chan School of Public Health. The return of a completed questionnaire was accepted by the IRB as implied informed consent. The study adhered to the Strengthening the Reporting of Observational Studies in Epidemiology (STROBE) reporting guideline.

We conducted a prospective study in the Nurses’ Health Study II between 2003 and 2017 among middle-aged females free of depression at baseline. Diet was assessed using validated food frequency questionnaires (FFQs) every 4 years. We estimated UPF intake using the NOVA classification,^2^ which groups foods according to the degree of their processing. In secondary analyses, we classified UPF into their components, including ultraprocessed grain foods, sweet snacks, ready-to-eat meals, fats and sauces, ultraprocessed dairy products, savory snacks, processed meat, beverages, and artificial sweeteners.^4^ We used 2 definitions for depression: (1) a strict definition requiring self-reported clinician–diagnosed depression and regular antidepressant use and (2) a broad definition requiring clinical diagnosis and/or antidepressant use.

We estimated hazard ratios (HRs) and 95% CIs for depression according to quintiles of UPF intake using Cox proportional hazards models, with adjustment for known and suspected risk factors for depression, including age, total caloric intake, body mass index (BMI; calculated as weight in kilograms divided by height in meters squared), physical activity, smoking status, menopausal hormone therapy, total energy intake, alcohol, comorbidities (eg, diabetes, hypertension, dyslipidemia), median family income, social network levels, marital status, sleep duration, and pain. In an exploratory analysis, we examined the association between changes in UPF consumption updated every 4 years with incident depression. All analyses were performed using 2-sided tests from SAS (version 9.4). Data were analyzed from September 2022 to January 2023.

## Results

Our cohort included 31 712 females, aged 42 to 62 years at baseline (mean [SD] age, 52 [4.7] years; 30 190 [95.2%] non-Hispanic White females). Participants with high UPF intake had greater BMI, higher smoking rates, and increased prevalence of comorbidities like diabetes, hypertension, and dyslipidemia and were less likely to exercise regularly. We identified 2122 incident cases of depression using the strict definition and 4840 incident cases using the broad definition. Compared with those in the lowest quintile of UPF consumption, those in the highest quintile had an increased risk of depression, noted for both strict definition (HR, 1.49; 95% CI, 1.26-1.76; *P* < .001) and broad definition (HR, 1.34; 95% CI, 1.20-1.50; *P* < .001) (Table). Models were not materially altered after inclusion of potential confounders. We did not observe differential associations in subgroups defined by age, BMI, physical activity, or smoking. In a 4-year lag analysis, associations were not materially altered (strict definition: HR, 1.32; 95% CI, 1.13-1.54; *P* < .001), arguing against reverse causation.

**Table.** Ultraprocessed Food Intake is Associated With Increased Risk of Depression in the Nurses’ Health Study II

Quintile	Cases, No.	Person-years	Age-adjusted HR (95% CI)^a^	Multivariate HR (95% CI)^b^	Multivariate HR (95% CI) with diet quality adjustment^c^
**Strict definition of depression** ^d^					
1 (<4 servings/day)	351	86 100	1 [Reference]	1 [Reference]	1 [Reference]
2 (4-5.3 servings/day)	397	86 623	1.10 (0.95-1.27)	1.11 (0.96-1.29)	1.10 (0.95-1.28)
3 (5.3-6.8 servings/day)	419	85 495	1.20 (1.04-1.38)	1.22 (1.05-1.41)	1.20 (1.04-1.40)
4 (6.8-8.8 servings/day)	429	86 273	1.20 (1.04-1.39)	1.23 (1.06-1.44)	1.22 (1.04-1.42)
5 (>8.8 servings/day)	526	86 101	1.49 (1.30-1.71)	1.52 (1.30-1.79)	1.49 (1.26-1.76)
*P* value for trend^e^	NA	NA	<.001	<.001	<.001
**Broad definition of depression** ^f^					
1 (<4 servings/day)	351	86 100	1 [Reference]	1 [Reference]	1 [Reference]
2 (4-5.3 servings/day)	397	86 623	1.05 (0.96-1.15)	1.06 (0.96-1.17)	1.06 (0.96-1.16)
3 (5.3-6.8 servings/day)	419	85 495	1.14 (1.04-1.25)	1.15 (1.05-1.27)	1.14 (1.04-1.26)
4 (6.8-8.8 servings/day)	429	86 273	1.17 (1.07-1.28)	1.20 (1.08-1.32)	1.18 (1.07-1.31)
5 (>8.8 servings/day)	526	86 101	1.34 (1.22-1.46)	1.37 (1.23-1.52)	1.34 (1.20-1.50)
*P* value for trend^e^	NA	NA	<.001	<.001	<.001

Abbreviations: HR, hazard ratio; NA, not applicable.

^a^ Adjusted for age.

^b^ Additionally adjusted for body mass index (calculated as weight in kilograms divided by height in meters squared), physical activity, smoking status, menopausal hormone therapy, total caloric intake, alcohol intake, comorbidities (history of diabetes, hypertension, dyslipidemia), median family income, social network levels, marital status, sleep duration, and pain.

^c^ Additionally adjusted for overall diet quality (defined by prudent diet pattern score).

^d^ Depression diagnosis by clinician and use of antidepressants.

^e^ Tests for linear trend (*P* value trend) were performed using the median value of each quintile of ultraprocessed foods consumption as a continuous variable in the regression model.

^f^ Depression diagnosis by clinician and/or use of antidepressants.

Next, we examined the association of specific UPF components with risk of depression. Comparing extreme quintiles, only artificially sweetened beverages (HR, 1.37; 95% CI, 1.19-1.57; *P* < .001) and artificial sweeteners (HR, 1.26; 95% CI, 1.10-1.43; *P* < .001) were associated with greater risk of depression and after multivariable regression (Figure). In an exploratory analysis, those who reduced UPF intake by at least 3 servings per day were at lower risk of depression (strict definition: HR, 0.84; 95% CI, 0.71-0.99) compared with those with relatively stable intake in each 4-year period.

**Figure.**
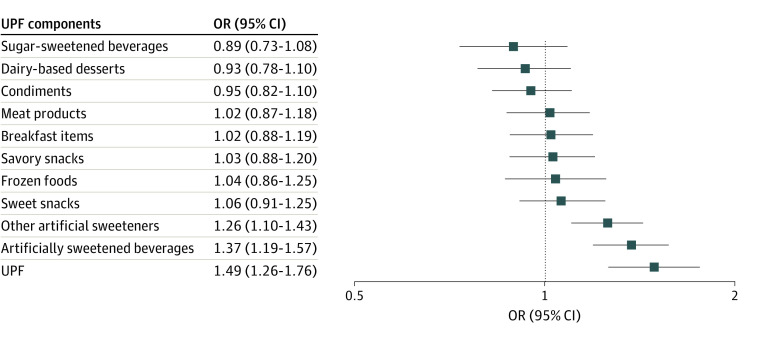
Ultraprocessed Foods (UPF) Components and Risk of Incident Depression OR indicates odds ratio. Comparing extreme quintiles of intake, artificially sweetened beverages, and artificial sweeteners were associated with greater risk of depression (strict definition) after multivariable regression.

## Discussion

These findings suggest that greater UPF intake, particularly artificial sweeteners and artificially sweetened beverages, is associated with increased risk of depression. Although the mechanism associating UPF to depression is unknown, recent experimental data suggests that artificial sweeteners elicit purinergic transmission in the brain,^5^ which may be involved in the etiopathogenesis of depression.^6^ Strengths of our study include the large sample, prospective design, high follow-up rate, ability to adjust for multiple confounders, and extensively validated dietary assessment tools. This study had limitations. The cohort primarily included non-Hispanic White females. Additionally, without structured clinical interviews, misclassification of the outcome may be considered.

## Notes

Supplement.
Data Sharing Statement

## References

[1] Adjibade M, Julia C, Allès B, . Prospective association between ultra-processed food consumption and incident depressive symptoms in the French NutriNet-Santé cohort. BMC Med. 2019;17(1):78. doi:10.1186/s12916-019-1312-y 30982472 PMC6463641

[2] Zheng L, Sun J, Yu X, Zhang D. Ultra-processed food is positively associated with depressive symptoms among United States adults. Front Nutr. 2020;7:600449. doi:10.3389/fnut.2020.600449 33385006 PMC7770142

[3] Gómez-Donoso C, Sánchez-Villegas A, Martínez-González MA, . Ultra-processed food consumption and the incidence of depression in a Mediterranean cohort: the SUN Project. 2020;59(3):1093-1103. doi:10.1007/s00394-019-01970-1 31055621

[4] Hang D, Wang L, Fang Z, . Ultra-processed food consumption and risk of colorectal cancer precursors: results from 3 prospective cohorts. J Natl Cancer Inst. 2023;115(2):155-164. doi:10.1093/jnci/djac221 36477589 PMC9905956

[5] Buchanan KL, Rupprecht LE, Kaelberer MM, . The preference for sugar over sweetener depends on a gut sensor cell. Nat Neurosci. 2022;25(2):191-200. doi:10.1038/s41593-021-00982-7 35027761 PMC8825280

[6] Szopa A, Socała K, Serefko A, . Purinergic transmission in depressive disorders. Pharmacol Ther. 2021;224:107821. doi:10.1016/j.pharmthera.2021.107821 33607148

