# Errors in the Table

**DOI:** 10.1001/jamanetworkopen.2023.41346

**Published:** 2023-10-18

In the Research Letter titled “Consumption of Ultraprocessed Food and Depression,”^1^ published September 20, 2023, there are numerical errors in the Table. There was a duplication in the case numbers and person-years for the broad definition of depression. This article has been corrected.^1^
