# Supplementary material for: Consumption of Ultraprocessed Food and Risk of Depression
Source: JAMA Netw Open. 2023 Sep 20;6(9):e2334770. doi: 10.1001/jamanetworkopen.2023.34770 (PMC10512104; doi:10.1001/jamanetworkopen.2023.34770)
Supplement: Supplement. — Data Sharing Statement [file jamanetwopen-e2334770-s001.pdf]

Data Sharing Statement

Samuthpongtorn. Consumption of Ultraprocessed Food and Risk of Depression. *JAMA Netw Open*. Published September 20, 2023. doi:10.1001/jamanetworkopen.2023.34770

Data
Data available: Yes
Data types: Deidentified participant data
How to access data: Access to the Nurses Health Study 2 data available upon request from Dr. Chan (achan@mgh.harvard.edu)
When available: With publication

Supporting Documents
Document types: None

Additional Information
Who can access the data: researchers whose proposed use of the data has been approved
Types of analyses: for any purpose
Mechanisms of data availability: with a signed data access agreement
Any additional restrictions: All SAS code is available at a github link.
